# Alveolar epithelial cell dysfunction and epithelial-mesenchymal transition in pulmonary fibrosis pathogenesis

**DOI:** 10.3389/fmolb.2025.1564176

**Published:** 2025-04-24

**Authors:** Caopei Zheng, Ling Zhang, Yuqing Sun, Yingmin Ma, Yulin Zhang

**Affiliations:** ^1^ Department of Respiratory and Critical Care Medicine, Beijing Youan Hospital, Capital Medical University, Beijing, China; ^2^ Laboratory for Clinical Medicine, Capital Medical University, Beijing, China; ^3^ Beijing Institute of Hepatology, Beijing Youan Hospital, Capital Medical University, Beijing, China; ^4^ Beijing Research Center for Respiratory Infectious Diseases, Beijing, China

**Keywords:** alveolar epithelial cell, epithelial-mesenchymal transition, molecular mechanisms, signaling pathways, pulmonary fibrosis

## Abstract

Pulmonary fibrosis (PF) is a progressive and lethal interstitial lung disease characterized by aberrant scar formation and destruction of alveolar architecture. Dysfunctional alveolar epithelial cells (AECs) play a central role in initiating PF, where chronic injury triggers apoptosis and disrupts epithelial homeostasis, leading to epithelial-mesenchymal transition (EMT). This dynamic reprogramming process causes AECs to shed epithelial markers and adopt a mesenchymal phenotype, fueling fibroblast activation and pathological extracellular matrix (ECM) deposition. This review systematically explores the multi-layered mechanisms driving AECs dysfunction and EMT, focusing on core signaling axes such as transforming growth factor-β (TGF-β)/Smad, WNT/β-catenin, NF-κB-BRD4, and nuclear factor erythroid 2-related factor 2 (Nrf2), which regulate EMT and fibroblast-ECM interactions. It also highlights emerging regulators, including metabolic reprogramming, exosomal miRNA trafficking, and immune-epithelial interactions. Furthermore, understanding these mechanisms is essential for developing targeted therapeutic strategies to modulate these pathways and halt or reverse fibrosis progression, offering critical insights into potential clinical treatments for PF.

## 1 Introduction

Pulmonary fibrosis (PF) is a chronic, progressive, interstitial fibrotic disease characterized by alveolar epithelial cells (AECs) damage, fibrotic cells proliferation, interstitial inflammation, and fibrosis, often manifesting together within the alveolar wall (Lederer and Martinez, 2018). Despite advances in understanding its pathogenesis, PF remains a devastating disease with limited treatment options and a poor prognosis (Martinez et al., 2017). Although antifibrotic agents such as pirfenidone and nintedanib are available, these therapies primarily slow disease progression rather than reverse fibrosis, underscoring the urgent need for more effective treatment strategies (Lederer and Martinez, 2018). Various factors, including recurrent infections, chronic inflammation, and environmental exposures, are known to contribute to the onset and progression of PF, the exact pathological mechanisms remain incompletely understood. In recent years, increasing evidence have indicated that AECs play a crucial role in the pathogenesis of PF (Long et al., 2024; Wang et al., 2022). Primarily responsible for maintaining the integrity of the alveolar barrier, AECs are among the first to respond to lung injury. Beyond their structural role, AECs also regulate barrier immunity, helping to maintain pulmonary microecological balance (Lee and Park, 2022; Zhang et al., 2017; Krausgruber et al., 2020). In the early stages of PF, damage or dysfunction in AECs disrupts the alveolar-capillary barrier. This disruption triggers a cascade of events, notably activating signaling pathways such as transforming growth factor-β (TGF-β)/Smad, NF-κB, and WNT/β-catenin, which collectively drive epithelial-mesenchymal transition (EMT) (Peng et al., 2020).

EMT is a biological process whereby AECs lose their characteristic polarity and cell-cell adhesion properties, gaining mesenchymal traits such as increased migratory capacity and resistance to apoptosis (Peng et al., 2020; Sgalla et al., 2018). In the context of PF, EMT contributes to the expansion of fibroblast and myofibroblast populations, cells that are key drivers of extracellular matrix (ECM) production and fibrosis progression (Jing et al., 2019). Increasing evidence suggests that EMT not only plays a mechanistic role in fibrogenesis but may also correlate with disease severity and treatment response, making it a potential biomarker or therapeutic target (Li et al., 2024; Jin et al., 2024). Despite these insights, the precise mechanisms by which AECs and EMT contribute to PF are not fully understood. Moreover, accumulating evidence suggests that that AECs are not merely passive victims of injury but actively participate in the fibrotic process through dysregulated signaling pathways that promote EMT and fibrosis (Wu et al., 2018). The interplay between AECs dysfunction and the microenvironment, including inflammatory mediators and oxidative stress, further complicates the progression of PF (Sauler et al., 2019; Shenderov et al., 2021).

Given the critical role of AECs dysfunction and EMT in fibrosis, a deeper understanding of these processes could provide novel therapeutic strategies to halt or reverse PF progression. This review aims to comprehensively explore the current understanding of AECs dysfunction and EMT in PF pathogenesis, with an emphasis on their molecular mechanisms and potential clinical implications. By bridging basic research with clinical perspectives, we hope to identify new opportunities for therapeutic advancements in PF management.

## 2 Inflammatory response and oxidative stress in alveolar epithelial cell

### 2.1 Cytokines in the inflammatory pathways of PF

The inflammatory response plays a pivotal role in the initiation and progression of PF. When the lungs are exposed to endogenous and exogenous factors, alveolar macrophages are activated and initiate inflammatory responses, promoting neutrophil infiltration and the release of pro-inflammatory cytokines such as tumor necrosis factor (TNF)-α, interleukin (IL)-1β, and IL-6 (Hu and Christman, 2019; Chen et al., 2022). These cytokines amplify the inflammatory cascade, leading to the recruitment of additional inflammatory cells, including eosinophils and lymphocytes, into the alveolar space. Prolonged inflammatory response leads to the production of various reactive oxygen species (ROS), which further cause damage to AECs and exacerbate the release of inflammatory mediators, creating a vicious cycle that perpetuates lung injury and fibrosis (Wu et al., 2018; Sauler et al., 2019).

Among the pro-inflammatory cytokines, interleukin (IL)-1β and IL-6 are particularly critical in driving AECs dysfunction and fibrosis. IL-1β, produced by activated macrophages and damaged AECs, acts as a key mediator of AECs injury and repair (Aumiller et al., 2013). It induces the expression of IL-6 in alveolar type 2 (AT2) epithelial cells, which in turn activates the signal transducer and activator of transcription 3 (STAT3) signaling pathway (Johnson et al., 2018). STAT3 is a central mediator of fibrosis, it can bind to the enhancer of the COL1A2 gene to enhance the transcriptional expression of type I collagen. Additionally, it promotes the survival and activation of fibroblasts and myofibroblasts, which are the key effector cells responsible for ECM deposition (Liu et al., 2025). A recent multi-omics analysis revealed that circ0066187, a pro-fibrotic metabolism-related factor, promotes the differentiation of fibroblasts into myofibroblasts by sponging miR-29b-2-5p and directly targeting STAT3 (Liu et al., 2025). Intervention targeting circ0066187 was shown to prevent the development of PF. Therefore, circ0066187 may serve as a metabolism-related therapeutic target for PF. Furthermore, IL-6 and STAT3 signaling are downstream effectors of the WNT/β-catenin pathway, which is aberrantly activated in PF and plays a crucial role in regulating inflammation and ECM deposition (Wang T. et al., 2024). Studies have shown that WNT/β-catenin signaling in AECs leads to the production of IL-1β and IL-6, creating a pro-fibrotic microenvironment that sustains TGF-β signaling and drives fibrosis progression (Aumiller et al., 2013). During fibrosis progression, dysregulated activation of the WNT/β-catenin signaling pathway disrupts β-catenin homeostasis. WNT receptor signaling induces the disassembly of the β-catenin degradation complex, preventing its proteasomal degradation (Li et al., 2021; Nusse and Clevers, 2017). As a result, β-catenin accumulates in the cytoplasm and translocates into the nucleus, where it interacts with T-cell factor (TCF) and lymphoid enhancer factor (LEF) to drive the transcription of pro-fibrotic genes, leading to aberrant fibroblast activation (Nusse and Clevers, 2017). Notably, studies have shown that β-catenin siRNA effectively inhibits WNT/β-catenin signaling and mitigates bleomycin (BLM)-induced PF in mice (Li et al., 2021).

In addition to IL-1β and IL-6, IL-17A has emerged as a critical cytokine in PF pathogenesis. IL-17A is primarily secreted by Th17 cells, a subset of CD4^+^ T cells that are induced by TGF-β, IL-6, IL-1β, and IL-23. IL-17A promotes EMT in AECs in a TGF-β-dependent manner, contributing to the expansion of fibroblast and myofibroblast populations (Park et al., 2023; Zhang, 2018). Elevated levels of IL-17A have been detected in the BALF and fibrotic lesions of PF patients, underscoring its role in disease progression (Celada et al., 2018). Targeting the IL-17A signaling pathway has shown promise in preclinical models, with studies demonstrating that IL-17A blockade attenuates bleomycin-induced fibrosis (Park et al., 2023). Moreover, theophylline, a drug commonly used for asthma, has been shown to inhibit TGF-β-induced IL-6 expression in AECs, thereby suppressing Th17 differentiation and offering a potential therapeutic strategy for PF (Park et al., 2023).

Another cytokine of interest in PF is IL-33, primarily secreted by damaged AT2 cells, which signals through its receptor IL-33R to mediate inflammatory responses (Yi et al., 2022; Takashima et al., 2025). Recent studies have highlighted the role of ubiquitin-specific protease 38 (Usp38) in negatively regulating IL-33R signaling via the autophagy–lysosome pathway. Usp38 deficiency exacerbates IL-33-induced pro-inflammatory responses, leading to increased inflammatory cell infiltration, collagen deposition, and α-SMA expression in mouse models of PF (Yi et al., 2022). Treatment with IL-33R neutralizing antibodies has been shown to alleviate these inflammatory and fibrotic changes, suggesting that targeting the IL-33/IL-33R axis may represent a novel therapeutic approach for PF (Yi et al., 2022).

The NF-κB signaling pathway is another critical mediator of inflammation in PF. Activation of NF-κB in AECs, particularly those with the *SFTPCI73T* mutation, leads to the secretion of inflammatory mediators such as granulocyte-macrophage colony-stimulating factor (GM-CSF), C-X-C motif chemokine ligand 5 (CXCL5), and matrix metalloproteinase-1 (MMP-1) (Alysandratos et al., 2021). These mediators further recruit inflammatory cells and contribute to ECM remodeling. Additionally, NF-κB activation is closely linked to ROS production, creating a feedback loop that perpetuates inflammation and fibrosis (Aumiller et al., 2013).

**FIGURE 1:**
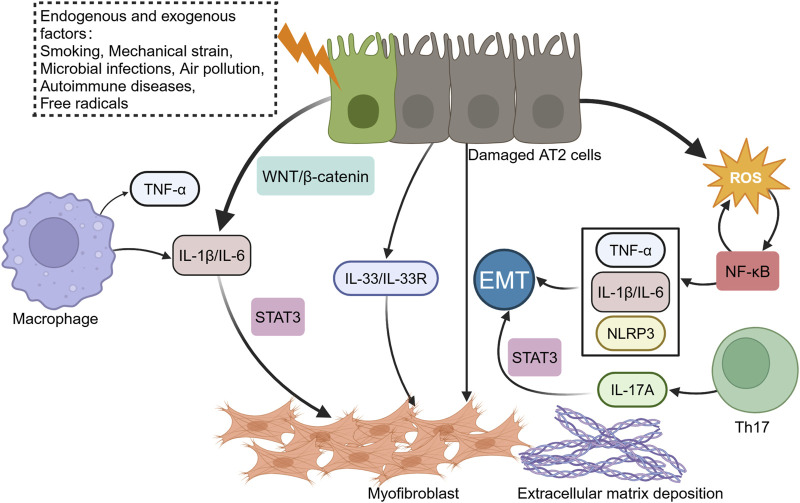
Interaction between inflammatory response and alveolar epithelial cell injury. When the lungs are invaded by endogenous or exogenous factors, alveolar macrophages trigger inflammation, releasing cytokines like tumor necrosis factor-α (TNF-α), interleukin (IL)-1β (IL-1β), and IL-6. Prolonged inflammatory responses lead to the production of various reactive oxygen species (ROS). Damaged alveolar type 2 (AT2) epithelial cells exacerbate the release of inflammatory mediators and ROS by activating the NF-κB and WNT/β-catenin signaling pathways, recruiting more pro-inflammatory cells and worsening extracellular matrix deposition. The WNT/β-catenin pathway induces IL-1β and IL-6 in AECs, while IL-17A promotes epithelial-mesenchymal transition (EMT) through STAT3 signaling pathway.

In summary, cytokines such as IL-1β, IL-6, IL-17, and IL-33 play central roles in the inflammatory pathways of PF. They drive AECs dysfunction, promote fibroblast activation, and sustain fibrotic responses through complex interactions with signaling pathways such as WNT/β-catenin, STAT3, and NF-κB. Understanding these mechanisms provides valuable insights into potential therapeutic targets for PF (Figure 1).

### 2.2 Oxidative stress, endoplasmic reticulum stress, and mitochondrial dysfunction in PF pathogenesis

**FIGURE 2:**
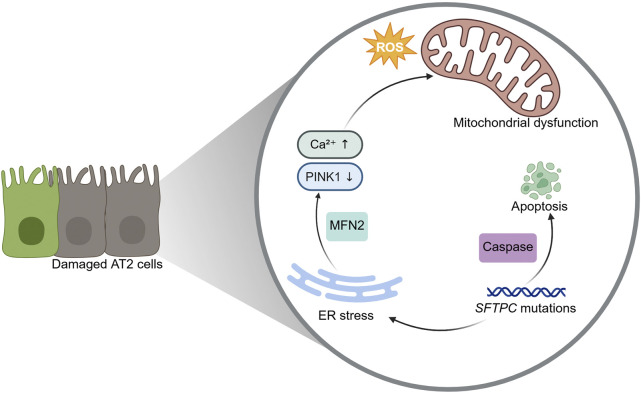
The imbalance of oxidative and reduction reactions in alveolar epithelial cells. Oxidative stress, endoplasmic reticulum (ER) stress, and mitochondrial dysfunction occur in alveolar type 2 (AT2) epithelial cells of pulmonary fibrosis patients. Mutations in the *SETPC* gene lead to ER stress and the activation of caspases. Mitofusin 2 protein (MFN2) facilitates Ca^2+^ transfer from the ER to mitochondria. ER stress also increases the mitochondrial apoptotic response by downregulating PTEN-induced putative kinase 1 (PINK1), ultimately causing mitochondrial swelling, loss of cell viability, and activation of pro-fibrotic responses.

The imbalance of oxidative and reduction reactions in AECs is a pathogenic factor in PF. Excessive ROS damage cellular macromolecules, leading to tissue injury and fibrosis (Todd et al., 2012). In IPF patients, oxidative stress, endoplasmic reticulum (ER) stress, and mitochondrial dysfunction are frequently observed in AT2 cells, contributing to AECs apoptosis (Shenderov et al., 2021). ER stress is usually caused by oxidative stress, genetic mutations, or viral infection, and is the result of excessive misfolding of proteins. To counteract ER stress, cells activate the unfolded protein response (UPR), a protective mechanism that enhances protein folding capacity and alleviates ER burden (Kelsen, 2016). In AT2 cells, mutations in the *SFTPC* gene hinder the proper processing and secretion of surfactant proteins, exacerbating ER stress and activating caspase-dependent apoptosis. Caspases, a family of proteases involved in cell growth, differentiation, and apoptosis, play a critical role in AECs loss during PF. Inhibition of caspase activity has been shown to attenuate fibrosis in preclinical models (Peng et al., 2020). Mitochondrial dysfunction is another hallmark of PF, characterized by impaired energy production and increased ROS generation. In IPF patients, mitochondrial dysfunction and oxidative stress are prevalent in AT2 cells further promoting AECs apoptosis (Shenderov et al., 2021; Zank et al., 2018). A key regulator of mitochondrial homeostasis is PTEN-induced putative kinase 1 (PINK1), which maintains mitochondrial morphology and function by facilitating the degradation of damaged mitochondria (Youle and Narendra, 2011). ER stress increases the mitochondrial apoptotic response by down-regulating PINK1 (Bueno et al., 2015). ER stress exacerbates mitochondrial dysfunction by downregulating PINK1 and increasing mitochondrial Ca^2+^ uptake through ER-mitochondrial coupling mediated by Mitofusin 2 (MFN2). While transient Ca^2+^ transfer enhances adenosine triphosphate (ATP) production, sustained ER stress leads to mitochondrial Ca^2+^ overload, swelling, and activation of pro-fibrotic responses (Bueno et al., 2015; Malhotra and Kaufman, 2011) (Figure 2).

### 2.3 Oxidative stress and NF-κB signaling pathway in silicosis and coal dust-induced PF

Silicosis, caused by prolonged exposure to silica dioxide, is a severe occupational lung disease characterized by persistent inflammation and progressive fibrosis (Hoy and Chambers, 2020; Franklin et al., 2016). Upon inhalation, silica particles, are engulfed by macrophages but resist degradation, triggering excessive production of ROS and activation of inflammatory signaling pathways, including NF-κB (Harijith et al., 2014). This leads to the release of pro-inflammatory cytokines such as IL-6 and TNF-α, which stimulate fibroblast recruitment, proliferation, and ECM deposition, ultimately driving PF (Sayan and Mossman, 2016). Histone deacetylase 10 (HDAC10) has emerged as a regulator of oxidative stress and inflammation in silicosis. HDAC10, as an upstream regulatory factor of ROS in silicosis, inhibits the accumulation of ROS by modulating protein acetylation, thereby suppressing the ROS/NF-κB signaling axis (Tian et al., 2023) (Figure 1).

Mechanistically, HDAC10 reduces ROS levels—a critical activator of NF-κB—and inhibits the phosphorylation of IκBα kinase β (IKKβ), IκBα, and p65, preventing NF-κB nuclear translocation and downstream inflammatory gene transcription (Tian et al., 2023). This antioxidant and anti-inflammatory role positions HDAC10 as a potential therapeutic target for silicosis-induced PF.

Similar to silica, coal dust nanoparticles (CD-NPs) induce oxidative stress, pulmonary inflammation, and EMT, contributing to PF (Blackley et al., 2018; Zhang et al., 2022). CD-NPs trigger ROS production, which activates NF-κB signaling and upregulates the transcription of inflammatory components (e.g., NLRP3, pro-IL-1β, and pro-IL-18) and caspase-1 (Qiu et al., 2019; An et al., 2019). Activated caspase-1 catalyzes the maturation of IL-1β and IL-18, amplifying the inflammatory response (Qiu et al., 2019). Insulin-like growth factor 1 (IGF1) has been implicated in ROS production and fibrotic progression (Pala et al., 2001). IGF1 promotes fibroblast proliferation and collagen synthesis, and its inhibition represents a potential therapeutic approach for PF (An et al., 2019). A positive feedback loop between inflammation and EMT in PF is mediated by the IGF1/IGF1R-ROS-AKT/GSK3β-NF-κB/NLRP3 signaling axis (Zhang et al., 2022). Specifically, IGF1/IGF1R-induced ROS activate AKT and inactivate GSK3β, leading to NF-κB/NLRP3 activation and subsequent inflammation and EMT (Zhang et al., 2022). Targeting this axis may alleviate CD-NPs-induced PF.

### 2.4 MAPK and Nrf2 signaling pathways in PF and oxidative stress regulation

The mitogen-activated protein kinase (MAPK) family, including JNK, ERK1/2, and p38 subgroups, plays a pivotal role in PF by mediating redox-sensitive responses to oxidative stress (Yan et al., 2016; van der Velden et al., 2016). These kinases are activated by ROS and drive fibrotic processes such as fibroblast proliferation, collagen deposition, and AECs apoptosis. Notably, reducing intracellular ROS levels suppresses MAPK pathways activation, highlighting their interdependence in PF progression (Ling et al., 2017). Studies have demonstrated that sarcodon aspratus fruiting polysaccharide (SAFP) exemplifies the therapeutic potential of targeting MAPK signaling. SAFP significantly inhibits JNK, ERK, and p38 phosphorylation in PF models, thereby reducing collagen deposition and ameliorating fibrosis (Dong et al., 2020).

Nuclear factor erythroid 2-related factor 2 (Nrf2) is a guardian against oxidative stress in PF. Under basal conditions, Nrf2 is sequestered in the cytoplasm by its inhibitor Keap1. Oxidative stress triggers Nrf2 phosphorylation, leading to Keap1 degradation via the Cul3-E3 ubiquitin ligase complex (Yan et al., 2017). Freed Nrf2 translocates to the nucleus, binds to antioxidant response elements (ARE), and activates transcription of cytoprotective genes such as heme oxygenase-1 (HO1) (Li et al., 2016). HO1 neutralizes superoxide radicals and enhances cellular resilience to oxidative damage. SAFP further amplifies this protective mechanism by upregulating Nrf2 and HO1 expression (Dong et al., 2020). MAPK signaling intersects with Nrf2 activation in PF. MAPK-mediated phosphorylation promotes Nrf2 nuclear translocation, while Nrf2 deficiency exacerbates ROS accumulation and fibrosis (Zhang et al., 2018). This bidirectional interaction underscores the importance of targeting both pathways for effective PF therapy. The scaffold protein p62 stabilizes Nrf2 by competitively binding Keap1 and promoting its autophagic degradation, thereby enhancing antioxidant responses (Liu et al., 2023). However, overexpression of spermatogenesis and oogenesis specific bhlh transcription factor 2 (Sohlh2) in AT2 cells disrupts p62/Keap1/Nrf2 signaling. This impairment leads to ROS overproduction, AECs apoptosis, and accelerated fibrosis (Liu et al., 2023).

## 3 EMT and PF

### 3.1 Molecular mechanisms of EMT and myofibroblast activation

**FIGURE 3:**
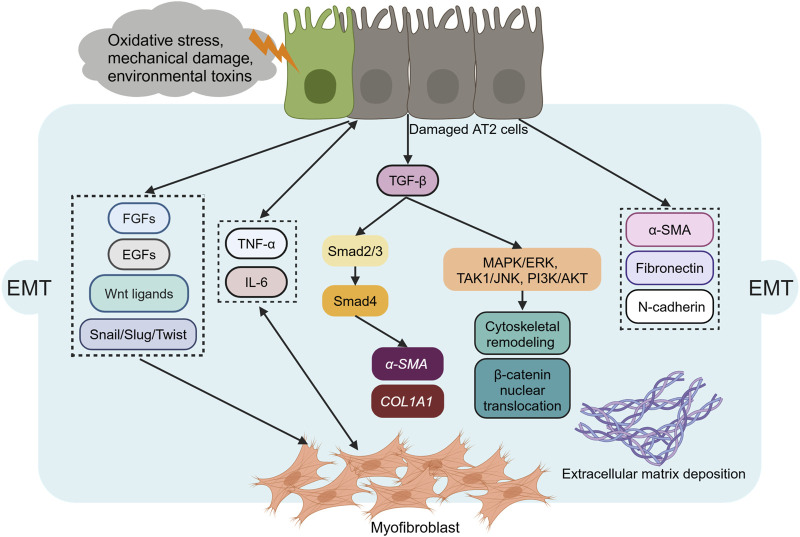
The molecular mechanisms of epithelial-mesenchymal transition. Alveolar type 2 (AT2) epithelial cells undergo epithelial-mesenchymal transition (EMT), transforming into myofibroblasts, with increased expression of N-cadherin, α-smooth muscle actin (α-SMA), fibronectin, and vimentin. Transforming growth factor-β (TGF-β), fibroblast growth factors (FGFs), epidermal growth factors (EGFs), and the Wnt/β-catenin signaling pathway can initiate EMT. EMT can also be initiated by extracellular transcription factors or activated signaling pathways. In the Smad-dependent pathway, activated TGF-β phosphorylates Smad 2/3 transcription factors in the cytoplasm, inducing the expression of target genes such as *α-SMA* and *COL1A1*. Non-Smad pathways such as the MAPK/ERK, TAK1/JNK, PI3K/AKT pathways, can also be activated to promote cytoskeletal remodeling and β-catenin nuclear translocation during the process of EMT.

EMT is a dynamic process wherein AECs lose epithelial markers (e.g., E-cadherin) and acquire mesenchymal traits (e.g., α-SMA, vimentin), contributing to myofibroblast accumulation in PF. Key transcriptional regulators of EMT include the Snail1/2, Zeb1/2, and Twist families, which repress epithelial genes and activate mesenchymal programs (Petukhov et al., 2019). Injury to quiescent AECs—triggered by oxidative stress, mechanical damage, or environmental toxins—initiates EMT through loss of apical-basal polarity and disruption of cell-cell junctions. Damaged AECs secrete profibrotic mediators such as TGF-β, fibroblast growth factors (FGFs), epidermal growth factors (EGFs), and Wnt ligands, which activate EMT transcription factors (Snail, Slug, Twist) and induce expression of mesenchymal markers (α-SMA, fibronectin, N-cadherin) (Petukhov et al., 2019; Skibba et al., 2020). These cells also produce aberrant ECM components, including alternative fibronectin (FN) splice variants, which signal through integrins to promote myofibroblast differentiation (Liu X. et al., 2021). TGF-β is a master regulator of EMT, acting through both Smad-dependent and non-Smad pathways. In the Smad-dependent pathway, TGF-β binding to its receptor phosphorylates Smad2/3, which complexes with Smad4 and translocates to the nucleus to activate transcription of pro-fibrotic genes (e.g., *COL1A1*, *α-SMA*) (Zhou et al., 2017). Non-Smad pathways such as the MAPK/ERK, TAK1/JNK, PI3K/AKT pathways can also be activated by TGF-β to promote cytoskeletal remodeling and β-catenin nuclear translocation during the process of EMT (Miao et al., 2022) (Figure 3). EMT-derived myofibroblasts secrete inflammatory cytokines (IL-6, TNF-α) and ECM proteins, creating a feed-forward loop that perpetuates epithelial injury and fibrosis (Torr et al., 2015). This vicious cycle is exacerbated by sustained ER stress in AECs, which amplifies FGF release and ECM remodeling (Skibba et al., 2020).

### 3.2 Molecular mechanisms of NF-κB-BRD4 complex in myofibroblast expansion

Chronic oxidative stress mediated by innate signaling pathways induces adaptive phenotypic switching in AECs, activating the EMT program. High-throughput RNA sequencing of normal human airway cells revealed significant overlap between the core regulatory network comprising 3,000 TGF-β-induced genes and the NF-κB signaling network, with NF-κB identified as the key driver of mesenchymal phenotypic transformation (Tian et al., 2018). Core EMT regulators include snail family zinc finger 1 (SNAI1), which promotes cellular dedifferentiation by suppressing epithelial adhesion proteins (Lamouille et al., 2014); zinc finger E-box binding homeobox 1 (ZEB1), a synergistic upregulator of TGF-β1 and vimentin expression (Lamouille et al., 2014); and twist family bhlh transcription factor 1 (TWIST1), an inducer of profibrotic factors such as periostin (Valenzi et al., 2023). Notably, bromodomain-containing protein 4 (BRD4) exerts multifunctional regulation in this process. This protein possesses intrinsic RNA polymerase kinase activity and modulates chromatin structure through atypical histone acetyltransferase (HAT) activity (Devaiah et al., 2016). TGF-β facilitates the assembly of the NF-κB-BRD4 complex, which leverages NF-κB sequence-specific DNA binding capacity to recruit BRD4 to promoter regions of innate inflammatory genes and EMT core regulators (Brown et al., 2014). This synergistic interaction ultimately leads to aberrant expression of collagen and fibronectin (FN), driving pathological processes including myofibroblast expansion, airway remodeling, and subepithelial basement membrane thickening.

### 3.3 Epigenetic regulation of EMT and fibroblast trans-differentiation in PF

Epigenetic modifications, which alter gene expression without DNA sequence changes, serve as a central mechanism driving fibrosis. HDACs and histone acetyltransferases (HATs) reciprocally regulate chromatin states through lysine residue modifications, with HDACs removing acetyl groups to promote chromatin condensation—a mechanism first identified in liver fibrosis where they enhance myofibroblast migration and ECM overproduction, subsequently validated by elevated HDAC expression in IPF tissues and isolated myofibroblasts (de Ruijter et al., 2003). In contrast, HATs such as p300 catalyze acetylation to facilitate chromatin relaxation, where p300 not only interacts with TGF-β1 to induce collagen promoter hyperacetylation but also undergoes nuclear accumulation under matrix stiffness to activate Smad2/3-dependent transcription of pro-fibrotic genes (*α-SMA, collagen, TIMPs*), collectively driving fibroblast activation and pathological ECM deposition (Dou et al., 2018). Hypoxia induces HDAC3-WDR5 complex formation, which suppresses epithelial genes while activating mesenchymal programs through chromatin remodeling (Lin and Wu, 2020). BRD4 functionally bridges EMT and fibroblast-to-myofibroblast trans-differentiation through bimodal regulatory mechanisms. It sustains epithelial-mesenchymal cell survival by activating anti-apoptotic pathways while simultaneously enhancing ECM biosynthesis through transcriptional elongation of fibrosis-associated genes (Devaiah et al., 2016). Furthermore, BRD4 acts as a critical amplifier of TGF-β signaling by orchestrating NOX4-dependent α-SMA expression and coordinating cytoskeletal contractility through Rho GTPase activation, thereby conferring mechanical motility to transitioning myofibroblasts (Ijaz et al., 2017). This dual capacity to regulate both phenotypic plasticity (EMT) and functional specialization (contractility/ECM deposition) establishes BRD4 as a pivotal node for therapeutic intervention in PF. DNA methyltransferase 1-associated protein 1 (DNMT1) collaborates with Snail to repress E-cadherin via promoter hypermethylation, triggering EMT or apoptosis in epithelial cells (Moen et al., 2015; Park et al., 2022).

### 3.4 Dysregulation of pulmonary surfactant system and exosomal signaling in EMT-Driven PF

In normal lungs, ATII cells are predominantly in a highly differentiated state and play crucial roles in the production, processing, and proper secretion of pulmonary surfactant. Pulmonary surfactant is critical for maintaining alveolar structure and preventing alveolar collapse during PF (Lutz et al., 2015). SP-B and SP-C are essential components of pulmonary surfactant. During the early stages of pulmonary fibrosis and the EMT process, deficiency of SP-B and SP-C leads to dysfunction of pulmonary surfactant, resulting in increased surface tension and alveolar collapse. Persistent and extensive alveolar injury triggers Notch1 activation, leading to hyperproliferation and dedifferentiation of ATII cells. This severely disrupts the surfactant system, induces elevated alveolar surface tension, and causes recurrent alveolar epithelial injury, forming a vicious cycle that ultimately contributes to pulmonary fibrosis (Wasnick et al., 2023). The CCDC59 protein acts as a transcriptional co-activator of SP-B and SP-C, specifically initiating their transcription. This enhances SP-B and SP-C expression, stabilizes alveolar structure, improves lung dynamic compliance, and ultimately alleviates BLM-induced PF (Zuo et al., 2024).

Identifying altered exosomal profiles and elucidating their roles in PF pathogenesis may provide critical insights for the therapeutic management of PF. Elevated levels of miR-21-5p have been observed in serum exosomes during both acute inflammatory and chronic fibrotic phases in mouse models of PF (Makiguchi et al., 2016). PF patients with higher miR-21-5p levels exhibit significantly worse prognoses within 30 months, suggesting its potential as a prognostic biomarker (Yang et al., 2022). In PF, fibroblast-derived exosomes show elevated levels of miR-23b-3p and miR-494-3p, which induce phenotypic changes in epithelial cells and correlate positively with disease severity (Kadota et al., 2020). Exosomal miRNA-328 from M2 macrophages enhances lung fibroblast proliferation and promotes PF progression (Yao et al., 2019; Kishore and Petrek, 2021). Conversely, macrophage-derived exosomes can inhibit PF progression by delivering the anti-fibrotic miR-142-3p to AEC and lung fibroblasts, thereby suppressing transforming growth factor-beta receptor 1 (TGFβ-R1) (Guiot et al., 2020).

### 3.5 EMT and immune microenvironment crosstalk

The interplay between EMT and the immune microenvironment represents a crucial yet underexplored axis in PF. During EMT, transitioning epithelial cells actively reshape the immune microenvironment through multifaceted mechanisms, beginning with the recruitment and polarization of immune cells. In the EMT process, transformed epithelial cells secrete cytokines such as CCL2, CSF-1, and IL-6, which induce monocyte polarization toward M2-type macrophages. Concurrently, in ER stress, GRP78 dissociates from its substrates, activating the UPR^ER^ pathway through PERK and IRE1α phosphorylation and ATF6 cleavage, which regulates macrophage differentiation to the M2 phenotype (Burman et al., 2018). These M2 macrophages subsequently secrete TGF-β, accelerating EMT and forming a pro-fibrotic positive feedback loop (Wang et al., 2020). Growing evidence also indicates that regulatory T cells (Tregs) can secrete TGF-β and activate the TGF-β/Smad pathway, shifting the Th1/Th2 balance while promoting fibroblast accumulation and EMT progression (Wang M. et al., 2024; Guo et al., 2020).

Simultaneously, EMT cells and immune cells collaboratively remodel the cytokine network. IL-6, secreted by both EMT cells and M2 macrophages, sustains the EMT phenotype via STAT3 phosphorylation (Johnson et al., 2018). TNF-α activates the NF-κB signaling pathway in fibroblasts, inducing MMP-9 release to disrupt the basement membrane and exacerbate fibrosis (Selvam et al., 2022). Additionally, IL-17A produced by Th17 cells enhances the EMT phenotype through STAT3 signaling. In both IPF patients and bleomycin-induced PF mice, PD-1^+^ Th17 cells exhibit elevated TGF-β/IL-17A expression (Celada et al., 2018). PD-1 regulates the transcription factor STAT3, and blocking the PD-1 pathway reduces IL-17A expression in Th17 cells, characterized by diminished pSTAT3 levels and significantly decreased collagen I production (Celada et al., 2018).

### 3.6 Impact of respiratory viral infections on PF

**FIGURE 4:**
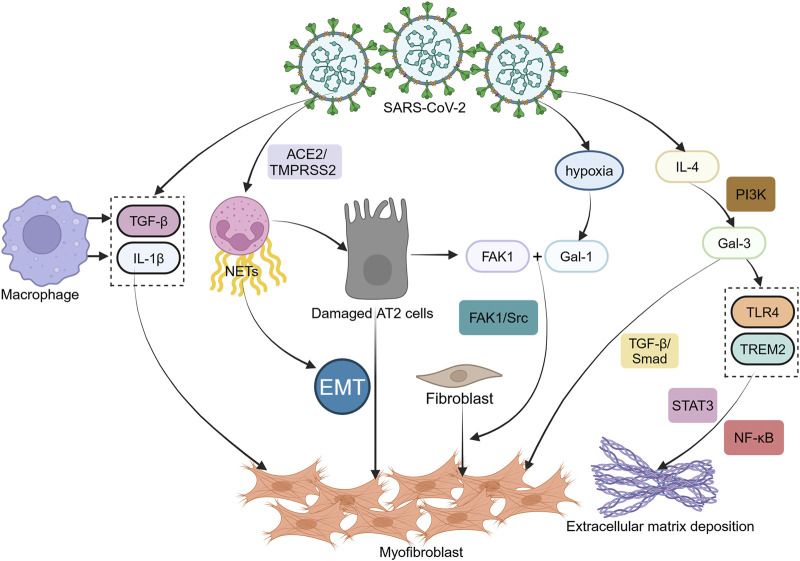
Severe acute respiratory syndrome coronavirus 2 infections and epithelial-mesenchymal transition. Following infection with severe acute respiratory syndrome coronavirus 2 (SARS-CoV-2), alveolar macrophages increase the secretion of cytokines like transforming growth factor-β (TGF-β), interleukin-1β (IL-1β), along with neutrophil extracellular traps (NETs), which promote epithelial-mesenchymal transition (EMT). Under hypoxic conditions, Galectin-1 (Gal-1) can interact with focal adhesion kinase-1 (FAK1) in alveolar type 2 (AT2) epithelial cells, accelerating the differentiation of fibroblasts into myofibroblasts. IL-4 stimulates the autocrine secretion of Gal-3 through the CD98-mediated phosphoinositide 3-kinase (PI3K) alternative activation pathway. Gal-3 activates Toll-like receptor 4 (TLR4) and triggering receptor expressed on myeloid cells 2 (TREM2), triggering NF-κB and signal transducer and activator of transcription 3 (STAT3) pathways that promote extracellular matrix deposition. It also activates the TGF-β1/Smad signaling axis, driving PF development.

Respiratory viral infections, including rhinovirus, respiratory syncytial virus (RSV), and SARS-CoV-2, exacerbate PF by inducing epithelial injury and dysregulated immune responses (Brasier, 2020; Hough et al., 2020). Coronavirus disease 2019 (COVID-19)-induced acute respiratory distress syndrome (ARDS) is characterized by hyperinflammation and elevated pro-fibrotic cytokines such as IL-6, IL-7, GM-CSF, MCP, and macrophage inflammatory protein-1α (MIP1α). Additionally, COVID-19 triggers ECM remodeling, with disease severity correlating with increased ECM components such as hyaluronic acid, type III collagen, and laminin (Ruan et al., 2020). Severe acute respiratory syndrome coronavirus 2 (SARS-CoV-2) infection amplifies alveolar macrophage-derived TGF-β and IL-1β, while neutrophil extracellular traps (NETs) promote EMT, further accelerating fibrosis (Pandolfi et al., 2021). Elevated levels of NETs were detected in the bronchoalveolar lavage fluid (BALF), plasma, and postmortem lung tissues of COVID-19 patients, along with an enhanced capacity of neutrophils to release NETs (Veras et al., 2020). By exposing extracellular DNA, myeloperoxidase (MPO), and histones, NETs promote apoptosis and fibrotic processes (Yadav et al., 2019). The release of NETs by neutrophils depends on the ACE2-TMPRSS2 pathway (Veras et al., 2020). Treatment of isolated neutrophils with either a neutralizing anti-human ACE2 antibody (αACE2) or the serine protease TMPRSS2 inhibitor camostat effectively suppressed SARS-CoV-2-induced NET release. Furthermore, one of the intracellular mechanisms driving NETosis involves the activation of peptidylarginine deiminase-4 (PAD4), as evidenced by reduced NETs release from blood neutrophils of COVID-19 patients following Cl-amidine treatment (a PAD4 inhibitor). Additionally, NETs may contribute to AECs damage during SARS-CoV-2 infection (Veras et al., 2020). Galectin-1 (Gal-1), a hypoxia-sensitive lectin, is upregulated in IPF and severe COVID-19. Under hypoxic conditions, Gal-1 binds focal adhesion kinase-1 (FAK1) in AECs, activating FAK1/Src signaling and driving fibroblast-to-myofibroblast differentiation (Kathiriya et al., 2017). This mechanism links viral-induced hypoxia to fibrotic progression (Markovic et al., 2022). Galectin-3 (Gal-3), structurally homologous to the SARS-CoV-2 spike protein N-terminal domain, facilitates viral entry by binding ACE2 receptors (Behloul et al., 2020; Garcia-Revilla et al., 2020). Post-infection, IL-4 stimulates Gal-3 autocrine secretion via CD98-PI3K signaling, which crosslinks CD98 on macrophages to amplify pro-fibrotic responses (Mackinnon et al., 2012). Furthermore, Gal-3 activates both Toll-like receptor 4 (TLR4) and triggering receptor expressed on myeloid cells 2 (TREM2), stimulating NF-κB and STAT3 inflammatory pathways that perpetuate ECM deposition (Burguillos et al., 2015; Boza-Serrano et al., 2019). Notably, Gal-3 also activates the TGF-β1/Smad signaling axis, thereby driving PF development (Du et al., 2025) (Figure 4).

## 4 Therapeutic perspectives

The rapidly evolving understanding of molecular pathways underlying PF, particularly AEC dysfunction and EMT, has significantly expanded the therapeutic landscape for this devastating disease. Building upon the mechanistic insights discussed throughout this review, several promising therapeutic strategies are emerging that target key pathological processes in PF. Recent advances have identified multiple targeted agents with significant antifibrotic potential.

STA-21, a novel nuclear and mitochondrial STAT3 inhibitor, has demonstrated efficacy in suppressing the secretory properties of fibroblasts, offering a potential approach to modulate the fibrotic microenvironment (Waters et al., 2019). Similarly, the natural compound Gracillin has shown potent STAT3 inhibitory activity by blocking STAT3 phosphorylation, preventing nuclear translocation of p-STAT3, and downregulating STAT3 target gene expression (Yang et al., 2021). Notably, the work by Mengyao Xie and colleagues has provided compelling evidence that Gracillin can effectively inhibit TGF-β1-driven p-STAT3 activation both *in vitro* and *in vivo*, resulting in significant attenuation of PF progression (Xie et al., 2023). The Wnt/β-catenin signaling pathway plays a critical role in mediating EMT (Yang et al., 2020) and represents another attractive therapeutic target, with betulinic acid emerging as a promising modulator. This compound has been shown to effectively attenuate the elevated expression of downstream Wnt/β-catenin target genes while inhibiting the nuclear accumulation of β-catenin (Li et al., 2021). In murine models of PF, administration of betulinic acid during the fibrotic phase resulted in substantial suppression of Wnt/β-catenin signaling pathway activity (Li et al., 2021). The quassinoid compound Bruceine A, derived from Brucea javanica, represents another innovative therapeutic approach through its targeting of Gal-3. By significantly reducing protein levels of both Smad2/3 and phosphorylated Smad2/3, Bruceine A effectively inhibits the TGF-β1/Smad signaling pathway, thereby demonstrating fibrosis-reversing potential (Du et al., 2025).

Emerging strategies also focus on addressing mitochondrial dysfunction and ER stress in PF pathogenesis. Thyroid hormone analogs have demonstrated particular promise by improving AECs survival through PINK1-dependent mechanisms, suggesting their potential as novel anti-fibrotic agents (Yu et al., 2018). Additionally, the downregulation of acyl-CoA synthetase short-chain family member 3 (ACSS3) in PF tissues has been identified as a key contributor to metabolic dysregulation, disrupting fatty acid oxidation and promoting anaerobic glycolysis in AECs. Importantly, restoration of ACSS3 expression has been shown to improve mitochondrial function, inhibit fibroblast activation, and reduce ECM deposition, collectively contributing to fibrosis alleviation (Jia et al., 2022; Wang L. et al., 2024). Microcystin-LR inhibits the UPR^ER^ pathway by reducing p-PERK, p-IRE1α, and cleaved ATF6, suggesting it may improve PF by suppressing CD206^+^ M2 macrophage differentiation (Wang et al., 2020).

The therapeutic potential of epigenetic modulation is exemplified by HDAC10, which has been demonstrated to reduce inflammation and PF through inhibition of the ROS/NF-κB signaling pathway (Tian et al., 2023). Another multifunctional agent, SAFP, has shown particular promise by simultaneously inhibiting TGF-β1/MAPK pathways while activating the protective Nrf2/HO1 axis (Dong et al., 2020). This dual mechanism of action positions SAFP as a particularly attractive candidate for PF treatment. Other approaches targeting oxidative stress include pharmacological Nrf2 inducers such as sulforaphane and p62 stabilizers, both of which have shown potential to counteract oxidative damage in PF (Liu P. et al., 2021). Furthermore, silencing Sohlh2 has emerged as a strategy to restore p62/Keap1/Nrf2 signaling and mitigate fibrotic progression (Liu et al., 2023).

Beyond these molecularly targeted approaches, treatments focusing on specific cell populations, including alveolar macrophages (Pentraxin-2) (Nakagawa et al., 2016) and AECs (PLN-74809) (Decaris et al., 2021), as well as novel CAR T cell therapies targeting fibroblast activation protein (FAP) (Rurik et al., 2022), are opening new avenues for IPF treatment. Future combination therapies that integrate immune modulators, anti-fibrotic agents, and cytokine inhibitors could offer synergistic benefits in halting or even reversing fibrotic processes.

## 5 Conclusion

In conclusion, AEC dysfunction and EMT are key drivers of PF. AEC injury disrupts lung homeostasis, triggering a cascade of events involving core signaling pathways like TGF-β/Smad, WNT/β-catenin, and NF-κB, which promote myofibroblast differentiation and ECM remodeling. Epigenetic changes and metabolic shifts lock AEC into a pro-fibrotic state, while microenvironmental interactions, including exosomes and immune cell crosstalk, further exacerbate fibrosis. A deeper understanding of these mechanisms is essential for the development of targeted therapeutic strategies that may halt or even reverse fibrosis progression, providing valuable insights for future PF treatments.
